# Psychometric validation of the addiction potential questionnaire among Iranian children aged 12–18 years using confirmatory factor analysis

**DOI:** 10.34172/hpp.025.44299

**Published:** 2025-07-15

**Authors:** Aboubakr Jafarnezhad, Haleh Ghaem Maralani, Ali Sahraian, Mohammad Aryaie, Jafar Hassanzadeh

**Affiliations:** ^1^Student Research Committee, Shiraz University of Medical Sciences, Shiraz, Iran; ^2^Non-Communicable Diseases Research Center, Department of Epidemiology, School of Health, Shiraz University of Medical Sciences, Shiraz, Iran; ^3^Research Center for Psychiatry and Behavioral Sciences, Shiraz University of Medical Sciences, Shiraz, Iran; ^4^Department of Primary Care and Public Health, School of Public Health, Imperial College London, London, UK; ^5^Department of Epidemiology, School of Health, Institute of Health, Research Centre for Health Sciences, Shiraz University of Medical Sciences, Shiraz, Iran

**Keywords:** Child, Factor analysis, Psychometrics, Questionnaires, Substance-related disorders, Validation

## Abstract

**Background:**

Drug addiction is a global social and health issue, with children being the most vulnerable group. This study aimed to validate the Addiction Potential Questionnaire for Iranian children aged 12 to 18 years using confirmatory factor analysis (CFA) and structural equation modeling (SEM).

**Methods:**

This cross-sectional study was conducted in 2023 on 600 students from Shiraz, Iran, using multi-stage sampling (stratified-cluster-simple random sampling). The Addiction Potential Questionnaire used in this study was originally developed by the research team to assess addiction potential among Iranian children. Construct validity was assessed through CFA, along with convergent and discriminant validity, and SEM. The reliability of the tool was calculated using Cronbach’s alpha, intra-class correlation coefficient, and composite reliability.

**Results:**

Results from CFA indicated a χ^2^/df ratio of 2.80. Additional fit indices also confirmed a good fit for the final model, including goodness-of-fit index (GFI=0.9), parsimonious comparative fit index (PCFI=0.74), and comparative fit index (CFI=0.8). Assessing convergent and discriminant validity revealed that all dimensions met acceptable standards. SEM results showed strong positive relationships among latent variables, with correlations of 0.82 between environmental-social-spiritual (ESS) and physical-psychological, and 0.98 between ESS and Other (*P*<0.001). The reliability indices for each factor were as follows: Other: Cronbach’s alpha (α)=0.46, Omega (composite reliability, CR)=0.43, Maximal reliability (Max(R))=0.44, intraclass correlation coefficient (ICC) [95% confidence interval (CI)]=0.74 (0.64–0.81), Akaike information criterion (AIC)=0.12. Family: α=0.70, CR=0.72, Max(R)=0.73, ICC [95% CI]=0.72 (0.61–0.79), AIC=0.24. Physical-psychological: α=0.80, CR=0.82, Max(R)=0.83, ICC [95% CI]=0.84 (0.78–0.88), AIC=0.35. ESS: α=0.61, CR=0.60, Max(R)=0.66, ICC [95% CI]=0.69 (0.58–0.78), AIC=0.13. Total Questionnaire: α=0.89, CR=0.85, Max(R)=0.86, ICC [95% CI]=0.88 (0.83–0.91), AIC=0.39

**Conclusion:**

The results indicated that the tool possesses good validity and reliability, making it a credible instrument for assessing addiction potential in Iranian children and for developing preventive programs.

## Introduction

 Addiction is a complex behavioral-psychological syndrome characterized by a strong compulsion to use substances, persistent consumption, and craving for re-use after cessation.^1^ According to the United Nations, addiction is considered a form of poisoning caused by the intake of natural or synthetic drugs, leading to serious consequences for both individuals and society.^2^

 Iran’s geographical location within the Golden Crescent has significantly contributed to the high prevalence of narcotics consumption in the country. United Nations statistics indicate that approximately 90% of global drug addicts and seized substances are concentrated in this region. This widespread addiction not only impedes individual personality development but also undermines family structures and social cohesion.^3^

 Children are among the most vulnerable groups, whether they are directly affected by addiction or living in families with addicted members. The decreasing age of addiction onset in Iran highlights the urgent need to address youth susceptibility to narcotics.^2,3^ According to a 2022 report by the United Nations Office on Drugs and Crime (UNODC), approximately 284 million individuals aged 15 to 64 worldwide had used drugs by 2020, representing a 26% increase over the previous decade.^4^

 Experts emphasize that measurement instruments for addiction must be culturally grounded and tailored to the target population to ensure validity.^5,6^ A comprehensive review of existing tools for assessing addiction tendencies among children aged 12 to 18 revealed a significant gap: there is no specific, standardized instrument designed for this age group.^7^ Current instruments have various limitations, including inadequate sample sizes, lack of specificity for subpopulations such as children or incarcerated women,^8,9^ incomplete reporting of validation indices, unclear questionnaire structures,^10-12^ and ambiguity regarding the number of questions in certain studies.^13-15^ While our previous study focused on establishing the face validity, content validity, and exploratory factor structure of the questionnaire, the present study advances this foundational work by employing confirmatory factor analysis (CFA) to rigorously test the measurement model, alongside comprehensive assessments of convergent and discriminant validity. Furthermore, we utilize structural equation modeling (SEM) to elucidate the underlying latent constructs and their interrelationships. This integrative psychometric approach not only reinforces the validity and reliability of the instrument but also provides a robust and theoretically grounded structural framework, thereby significantly extending and deepening the insights gained from our earlier research. Given the critical importance of mitigating the adverse consequences of addiction among children, there is a pressing need to develop and psychometrically evaluate a standardized questionnaire specifically designed for assessing addiction tendencies in Iranian children. This need is further underscored by community priorities outlined by the “Drug Control Headquarters”.^16^ The present study aims to fill this gap by providing a validated instrument capable of measuring addiction susceptibility, screening at-risk children, and facilitating timely referral to appropriate services.

## Methods

###  Study Design and Setting

 The study was conducted among students aged 12 to 18 years attending first and second cycle girls’ and boys’ schools across the four educational districts of Shiraz, Iran. This sampling approach ensured representation of diverse educational regions within the city. Further methodological details regarding the selection of these schools and participants are provided in the methodology section.

###  COSMIN-Based Psychometric Evaluation of the Study

 This study has been prepared after undergoing all psychometric evaluation stages according to the Consensus-based Standards for the selection of health Measurement Instruments (COSMIN) checklist, ensuring its validity and reliability.^17^

###  Sampling Procedure

 In this study, a multi-stage sampling method combining stratified, cluster, and simple random sampling was employed to ensure a representative sample of high school students in Shiraz. Initially, stratified random sampling was conducted based on three criteria: educational district (four districts in Shiraz), gender (male and female), and school level (first and second secondary levels), resulting in 16 distinct strata. All four educational districts were included to capture the full geographical and socioeconomic diversity of the city. Within each stratum, schools served as clusters, and a list of eligible schools was obtained from the Shiraz Department of Education. Subsequently, schools were randomly selected from each list using simple random sampling methods. This approach ensured proportional representation across districts, genders, and school levels, thereby enhancing the generalizability and validity of the findings while minimizing selection bias.

 Although a multi-stage sampling design combining stratified random sampling and cluster sampling was employed, adjustment for the design effect was not performed. This decision was based on the stratification by educational district, gender, and school level, which reduces sampling variance, the random selection of relatively small clusters (schools) within strata, and the expectation of low intra-cluster correlation for the measured variables. Therefore, the design effect was considered negligible and did not warrant adjustment in the analyses.

###  Data Collection Process

 The data collection was carried out in two phases. In the first phase, 400 students were selected for exploratory factor analysis (EFA), and in the second phase, 600 students were recruited for CFA and SEM. The total sample size of 1000 students was determined based on recommendations by Keller and Kelvin, who suggest a minimum of 5 to 10 participants per questionnaire item.^5,6^ Considering the final questionnaire contained 30 items, the sample size range was between 150 to 300; thus, the larger sample was chosen to ensure robustness and account for potential dropouts.^18^

###  Instrument Description

 The research variables included age, gender, education level, addiction potential, educational stage, school grade, and educational district. Inclusion criteria for the study were: students residing in Shiraz who are currently enrolled in either the first or second year of high school. Exclusion criteria included lack of cooperation and unwillingness to participate in the study. This research consisted of three phases.

###  Phase One: Initial Development of Questions, Assessment of Face Validity, Content Validity, Exploratory Factor Analysis, and Reliability

 This section has been published in previous articles^18^ and is briefly discussed here to aid in the understanding of the current paper. In the first phase, the initial stages of developing the questionnaire items were carried out. This involved a review of existing literature in both Persian and English databases and websites such as PubMed, Magiran, Google Scholar, SID, and Qolbank using keywords like “Addiction Potential Questionnaire” and “Potential Questionnaire on Iranian Population,” employing MeSH terms and combining synonyms with operators such as AND, OR, and NOT. After collecting articles and questionnaires through a systematic review,^7^ initial questions were gathered based on expert opinions, literature review, and questionnaire analysis. Subsequently, a pool of items was formed. Relevant questions were then designed and drafted. Once the questions were finalized, face validity was assessed quantitatively by 32 participants and qualitatively by 32 participants along with 15 experts. Following this, content validity was evaluated qualitatively and quantitatively by 15 experts from across the country. After designing the questionnaire and assessing face validity, reliability was evaluated through Cronbach’s alpha and intra-class correlation among participants. Following revisions made by participants and experts, a secondary version of the questionnaire was developed. Construct validity was then calculated using EFA. The construct validity was assessed through EFA using maximum likelihood Methods and Varimax rotation among 400 students.^19^ To evaluate the appropriateness of the sample size and model, the Kaiser-Meyer-Olkin (KMO) test and Bartlett’s test of sphericity were utilized. Additionally, the number of factors was determined based on eigenvalues greater than one, total variance explained (TVE), and scree plot analysis.^19,20^

###  Phase Two: Assessment of Construct Validity

 In the second phase, construct validity was measured using CFA, as well as convergent and discriminant validity. To evaluate the structural factors, CFA was conducted using the maximum likelihood method and the most common goodness-of-fit indices among 600 samples. The model fit was assessed based on the chi-square/degrees of freedom ratio (χ^2^/df), goodness-of-fit index (GFI), comparative fit index (CFI), incremental fit index (IFI), root mean square error of approximation (RMSEA), parsimonious comparative fit index (PCFI), and parsimonious normed fit index (PNFI).^5,6^

###  Convergent and Discriminant Validity

 To evaluate the convergent validity of the questionnaire, correlation analyses were conducted between each item and its corresponding hypothesized construct. A correlation coefficient greater than 0.40 was considered indicative of acceptable convergent validity, demonstrating that items adequately represent their intended factors. Discriminant validity was assessed by comparing the correlation of each item with its own construct against its correlations with other constructs. Discriminant validity was confirmed when an item’s correlation with its hypothesized factor was higher than its correlations with other dimensions or scales, indicating that each item uniquely measures its intended construct without excessive overlap. This approach aligns with established psychometric practices and allows for clear interpretation of construct validity.^21^

###  Phase Three: Structural Equation Modeling

 In this phase, SEM was conducted to examine the hypothesized relationships among latent variables. SEM allows for the simultaneous analysis of multiple equations and provides a comprehensive understanding of the complex interplay between latent constructs and their observed indicators. Specifically, SEM was used to evaluate the relationships between latent (unobserved) variables and observed variables, assessing how well the observed indicators represent the underlying constructs. CFA and SEM are integrated components of the same analytical framework rather than separate processes.^6,21^

###  Reliability

 Reliability was assessed through internal consistency using the following indices: Cronbach’s alpha (Alpha), intraclass correlation coefficient (ICC) was calculated using a two-way mixed-effects model (ICC(3,1)) with absolute agreement definition. This model was chosen because the same participants completed the questionnaire twice over a two-week interval, and we aimed to assess the absolute agreement between the repeated measurements. The single measurement type was selected to evaluate the reliability of individual scores at each time point. This approach follows established guidelines for test-retest reliability assessment.^6^ Additionally, maximum reliability (MaxR(H)), McDonald’s omega coefficient (Omega), composite reliability (CR), and average inter-item correlation (AIC) were calculated to provide a comprehensive evaluation of the measurement tool’s reliability.^5,6^

###  Multivariate Normality and Outliers

 Univariate distribution was assessed for outliers, skewness, and kurtosis, while multivariate distributions were evaluated for normality and multivariate outliers. Univariate distribution for normal data was examined with skewness ( ± 3) and kurtosis ( ± 7).^6,22^ Multivariate normality was initially evaluated using Mardia’s multivariate skewness coefficient; however, considering potential limitations of this measure, more emphasis was placed on skewness and kurtosis indices as primary indicators of normality. Multivariate outliers were identified using Mahalanobis distance, where items with a squared Mahalanobis distance exceeding the critical value at *P* < 0.001 were considered outliers.^6^ Upon detecting deviations from normality or outliers, data were carefully examined to assess their potential impact on the analysis. Since no substantial issues were identified, all data were retained for final analyses.

###  Statistical Analyses

 Statistical methods for analysis included descriptive and analytical indices, Pearson correlation coefficient, EFA, Cronbach’s alpha, ICC,goodness-of-fit indices, and more. Construct validity was assessed using maximum likelihood estimation in CFA. The assumptions of this method, including normal distribution of data, independence of observations, adequate sample size, existence of correlations among variables, and absence of outliers, were examined prior to analysis to ensure validity of the results.^5,6,22^

 For the related analyses, IBM SPSS Statistics for Windows, Version 26.0 (IBM Corp., Armonk, NY, USA) was used. For CFA and SEM, IBM SPSS Analysis of Moment Structures (AMOS) software, Version 26.0 (IBM Corp., Armonk, NY, USA) was employed.^6^

## Results

###  Descriptive Results

 In this study, 600 students participated, with a mean age of 15.10 (SD 1.92) years. The minimum age of the students was 12 years, and the maximum age was 18 years. Fifty percent (300 participants) were male. The number of students in each district was 150 (25%), and 50% (300 students) were enrolled in the first year of high school (Tables 1 and 2).

**Table 1:** Description of quantitative variable of participants

**Variable**	**Mean±SD**	**Min**	**Max**
Age	15.39 ± 1.94	12	18

**Table 2:** Description of qualitative variables of participants

**Variable**	**Category**	**Frequency (n=400)**	**Percent**
Gender	Girls	300	50
	Boys	300	50
Education district	1	150	25
	2	150	25
	3	150	25
	4	150	25
Study course	1st	300	50
	2nd	300	50

###  Findings of Phase One

 In Phase One,^18^ we developed a 30-item questionnaire based on prior qualitative and quantitative face validity assessments, which were confirmed by expert review. Content validity indices (CVR > 0.49, CVI > 0.79) indicated satisfactory content validity. EFA revealed a four-factor structure—Physical-Psychological, Family, environmental-social-spiritual (ESS), and Other—explaining 40% of the variance, with adequate sampling adequacy (KMO = 0.88) and significant Bartlett’s test (*P* < 0.001). Reliability analyses demonstrated strong internal consistency and stability (Cronbach’s alpha = 0.87, ICC = 0.88) for the overall instrument.^18^

 Prior to conducting the statistical analyses, key assumptions including normality and absence of outliers were assessed using appropriate diagnostic tests (e.g., Shapiro-Wilk test, boxplots). The results indicated that all assumptions were satisfactorily met, supporting the validity of the applied models.

 In this phase, the factor structure obtained from EFA^18^ was evaluated and validated through CFA using the maximum likelihood estimation method, based on data from 600 students. The model fit index yielded a chi-square value of χ^2^ = 11117.157 (*P* < 0.001, df = 399), and the ratio of χ^2^/df was 2.80. Additionally, other model fit indices were calculated, and the values of all indices confirmed the good fit of the final model (Table 3).

**Table 3:** Model fit indices for confirmatory factor analysis (CFA)

**Index**	**Computed index**	**Acceptable range**
Chi-square/degrees of freedom ratio (χ^2^/df)	2.80	< 5
Goodness-of-fit index (GFI)	0.90	Close to 1
Comparative fit index (CFI)	0.80	Close to 1
Incremental fit index (IFI)	0.80	Close to 1
Root mean square error of approximation (RMSEA)	0.05	< 0.08
Parsimonious comparative fit index (PCFI)	0.74	> 0.50
Tucker-Lewis index (TLI)	0.92	Close to 1
Standardized root mean square residual (SRMR)	0.07	< 0.1

 As presented in Table 3, the questionnaire demonstrated satisfactory convergent and discriminant validity across all factors. The range of item-to-factor correlations (corrected for overlap) exceeded the 0.40 threshold for convergent validity in most cases, with scaling success rates ranging from 84% to 100% across subscales. Discriminant validity was supported by the finding that item correlations with their hypothesized constructs were consistently higher than correlations with other factors, with scaling success rates between 95% and 100%. These results confirm that the questionnaire items effectively measure their intended constructs and maintain distinctiveness from other dimensions, thereby supporting the overall construct validity of the instrument (Table 4).

**Table 4:** Convergent and discriminant validity of factors in the Addiction Potential Questionnaire (n = 600)

**Factor**	**No. items**	**Mean ± SD**	**Convergent validity**^a^		**Discriminant validity**^b^	
			**Range of correlation**	**Scaling success (%)**	**Range of correlation**	**Scaling success (%)**
Other^c^	5	13.02 ± 6.68	0.47-0.62	5/5 (100)	0.18-0.34	15/15 (100)
Family	6	13.39 ± 4.37	0.41-0.75	5/6 (84)	0.17-0.53	17/18 (95)
Physical-Psychological	8	23.76 ± 7.43	0.56-0.75	8/8 (100)	0.22-0.52	24/24 (100)
ESS	11	27.13 ± 6.05	0.42-0.58	11/11 (100)	0.17-0.56	32/33 (97)

ESS, Environmental-social-spiritual. ^a^Number of correlation between items and hypothesized scale corrected for overla*p*≥ 0.4/ total number of convergent validity.^21^ ^b^Number of convergent correlations significantly higher than discriminant correlations/Total number of correlations.^21^ ^c^Other: This factor includes items that did not fit into the family, physical-psychological, or environmental-social-spiritual factors and covers a range of diverse aspects.

 Additionally, the assumptions of SEM were examined, including independence of observations, adequate sample size, existence of correlations between latent and observed variables, absence of outliers, correct model specification, and logical relationships between latent and observed variables. the results indicated that all assumptions were satisfactorily met, supporting the validity of the applied models. The results of the correlation analysis among the latent variables in SEM indicate strong positive relationships between these variables. Specifically, the correlation between ESS and physical-psychological was 0.82, demonstrating a strong connection between these two variables (*P* < 0.001). Additionally, the correlation

 of ESS with Other was recorded at 0.98 (*P* < 0.001), and with Family at 0.78 (*P* < 0.001), both indicating significant and close relationships. Other notable correlations include the relationship between ‘other’ and ‘physical-psychological’ (0.87), (*P* < 0.001) and between ‘physical-psychological’ and ‘family’ (0.85), (*P* < 0.001). These results highlight the existence of significant and strong relationships among the latent variables, emphasizing the importance of considering these relationships in efforts to better understand addiction tendencies (Table 5).

**Table 5:** Correlations between latent variables in structural equation modeling (SEM)

**Correlation between latent variables**	**Estimate**	***P*** ** value**
ESS < -- > Physical-Psychological	0.82	*
ESS < -- > Other	0.98	*
Other < -- > Physical-Psychological	0.87	*
Other < -- > Family	0.85	*
ESS < -- > Family	0.78	*
Physical-Psychological < -- > Family	0.86	*

ESS, Environmental-social-spiritual. * *P* < 0.001

 The results of the standardized regression weights analysis in SEM indicate significant relationships between the observed variables and the latent variables. The latent factors in the model are composed of specific observed items as follows (Table 6):

**Table 6:** Composition of latent factors and their corresponding items

**Latent factor**	**Items included**
ESS	Q1 to Q4, Q7, Q15 to Q17, Q22, Q29, Q30
Physical-psychological	Q6, Q11, Q12, Q18, Q19, Q23 to Q25
Family	Q9,Q10, Q20, Q21, Q27, Q28
Other*	Q5, Q8, Q13, Q14, Q26

ESS, Environmental-social-spiritual. * Other: This factor includes items that did not fit into the family, physical-psychological, or environmental- social-spiritual factors and covers a range of diverse aspects.

**Figure 1:**
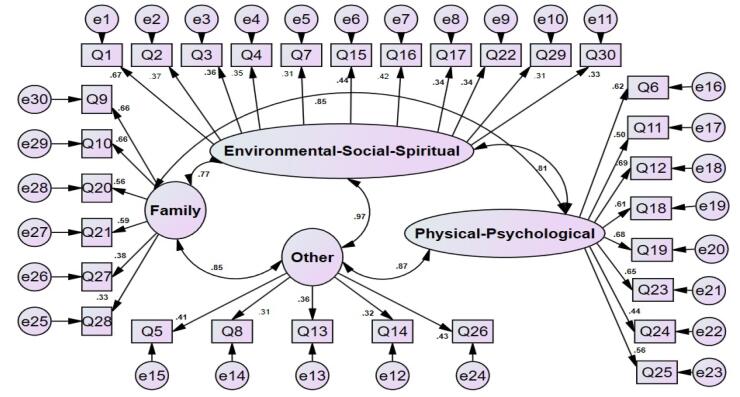
The factor named “Other” includes the items that were not loaded on the Family, Physical-Psychological, or Environmental-Social-Spiritual (ESS) factors, as the items represented diverse aspects; ESS model fit indices: CFI = 0.80, TLI = 0.92, RMSEA = 0.05, SRMR = 0.07.

 For the ESS variable, question Q15 with a regression weight of 0.44 demonstrates a strong impact of this variable on the quality of care. Regarding the physical-psychological factor, question Q19 shows a significant effect with a weight of 0.68. Additionally, for the family factor, question Q10 with a regression weight of 0.66 clearly confirms a positive relationship. Finally, for the Other factor, question Q14 with a weight of 0.32 represents the influence of this variable on the outcomes. These findings confirm the existence of significant positive relationships among the variables and strengthen the credibility of the model (Figure 1).

###  Reliability

 internal consistency of the entire questionnaire was 0.87, which indicates the strong correlation of the entire instrument. Also the ICC of the whole instrument was 0.88, which indicates a good to excellent agreement between domains and items. It was determined that the factors under investigation exhibited acceptable and desirable reliability. Specifically, the “physical-psychological” factor showed a Cronbach’s alpha of 0.80, an Omega (CR) of 0.82, and an ICC of 0.84 (95% CI: 0.78-0.88), indicating strong internal consistency and excellent agreement among the items. Additionally, the “family” factor demonstrated a Cronbach’s alpha of 0.70 and an ICC of 0.72 (95% CI: 0.61-0.79), reflecting moderate to strong internal consistency. These results suggest that the aforementioned factors effectively measure the intended constructs and possess high validity (Table 7).

**Table 7:** Reliability indices results in subscales and the overall questionnaire

**Factors**	**No. items**	**Alpha **	**ICC (95% CI)**	***P*** ** value**	**Max(R)**	**Omega**	**CR**	AIC*
Other	5	0.46	0.74 (0.64-0.81)	< 0.001	0.44	0.49	0.43	0.12
Family	6	0.70	0.72 (0.61-.0.79)	< 0.001	0.73	0.77	0.72	0.24
Physical-Psychological	8	0.80	0.84 (0.78-0.88)	< 0.001	0.83	0.85	0.82	0.35
ESS	11	0.61	0.69 (0.58-0.78)	< 0.001	0.66	0.63	0.60	0.13
Total	30	0.89	0.88 (0.83-0.91)	< 0.001	0.86	0.87	0.85	0.39

Abbreviations: ESS, environmental-social-spiritual;Alpha, Cronbach's alpha; ICC, intraclass correlation coefficient; MaxR (H), maximum reliability; Omega, McDonald’s omega coefficient; CR, composite reliability; AIC, average inter-item correlation. Note: "Other" factor includes items that did not load on the family, physical-psychological, or ESS factors and represent diverse aspects, *The ideal range of average inter-item correlation is 0.10 to 0.50; less than this, and the items are not well correlated and don’t measuring the same construct or idea very well (if at all). More than 0.50, and the items are so close as to be almost repetitive.^6,22^

## Discussion

 The results of this study indicate the desirable validity and reliability of the addiction potential questionnaire for Iranian children aged 12 to 18 years, utilizing various validation methods. The analyses performed include assessments of face validity, content validity, construct validity (EFA and CFA, convergent and discriminant validity), reliability, and SEM. These analyses clearly demonstrate that the latent variables, particularly ESS factors, family, and Physical-Psychological factors, have significant impacts on one another (*P*< 0.001). The calculated regression weights and covariance’s indicate strong positive relationships among these variables. Furthermore, the regression weights reveal that the family variable plays a key role in preventing addiction tendencies, particularly highlighted by high regression weights for questions Q9 (0.66) and Q10 (0.65).

 In a study conducted by Jurk et al titled “Personality and substance use: psychometric evaluation and validation of the Substance Use Risk Profile Scale (SURPS) in English, Irish, French, and German adolescents,” the authors reported strong internal consistency for the SURPS (Cronbach’s alpha coefficients ranging from 0.78 to 0.86 across subscales) and robust factorial validity, with most factor loadings exceeding 0.50. Their CFA yielded good model fit indices (CFI = 0.93, RMSEA = 0.05), supporting the multidimensional structure of the scale. Jurk et al also emphasized the predictive role of personality traits in adolescent substance use risk, highlighting the importance of individual differences in prevention.^23^ Consistent with these findings, the present study applied a comprehensive psychometric approach—including exploratory and confirmatory factor analyses, reliability assessment, and SEM—to validate the addiction tendency questionnaire for children. The questionnaire demonstrated excellent internal consistency (Cronbach’s alpha = 0.89), and CFA indicated satisfactory model fit (CFI = 0.80, TLI = 0.92, RMSEA = 0.05). Notably, regression weights for family-related items were particularly high (e.g., Q9 = 0.658, Q10 = 0.655), underscoring the central role of family in shaping addiction tendencies. The use of SEM in the current research enabled a more nuanced understanding of the interplay among latent variables, representing a methodological advancement over studies relying solely on CFA. Collectively, these results reinforce the necessity of multidimensional, context-sensitive assessment tools with robust psychometric properties (such as high Cronbach’s alpha, strong factor loadings, and good model fit indices) for accurately identifying and addressing substance use risk in youth.

 Our findings indicate that the family variable has a significant impact on addiction tendencies, aligning with previous studies.^24^ Specifically, the high regression weights for questions Q9 (0.658) and Q10 (0.655) highlight the key role of family in preventing addiction tendencies. These findings are consistent with research by Khalil et al,^25^ which emphasize the importance of family cohesion and parent-child attachment. Additionally, the results from Tarekegn et al suggest that family support can influence the quality of life of substance-using youth.^26^ However, discrepancies with other studies, such as Tam’s research that focuses on risk factors like academic stress and peer influence, may stem from differences in family structure or the type of community examined.^27^ Furthermore, our results align with study by Clark and Nguyen, which explore the role of familial factors in substance use.^28^ Ballester et al also emphasize the importance of family dynamics and parenting skills in preventing substance abuse.^29^ Ultimately, these findings underscore the significance of emotional and social support from families in protecting adolescents against social and peer pressures, consistent with study by Zeinali.^8^

 The study by Moghanloo and Valivand used SEM to examine the relationship between the Big Five personality traits, resilience, and addiction tendency. While their 38-item instrument demonstrated acceptable reliability (Cronbach’s alpha), the psychometric evaluation was limited, lacking convergent and discriminant validity assessments and relying primarily on CFA. Their findings indicated that four personality traits indirectly influenced addiction tendency through resilience.^13^ In contrast, the present study employed a comprehensive, multi-step psychometric approach—including assessments of face, content, convergent, and discriminant validity, as well as exploratory and confirmatory factor analyses, reliability indices, and SEM. This robust methodology confirmed the instrument’s reliability and validity and identified three key latent constructs: ESS factors, family, and physical-psychological factors, with family emerging as the strongest protective factor. Compared to previous research focusing on individual traits,^13^ our findings highlight the central role of environmental and familial factors in preventing addiction tendency among children. Overall, the developed instrument offers a more rigorous psychometric foundation and provides a valuable tool for designing targeted, evidence-based prevention programs.

 Moreover, Wagner et al linked decreased parental supervision to substance use, reinforcing the critical role of families in addiction prevention.^30^ However, Alhyas et al indicated that low parental supervision is a risk factor for substance use, while our findings emphasize the importance of positive family functioning. This discrepancy may arise from cultural or social differences.^31^

 The physical-psychological factor in this study had a significant impact on addiction tendencies, with regression weights for questions Q19 (0.683) and Q12 (0.691) indicating this influence. These findings align with the results of Whitesell et al, which show that the psychological status of adolescents is significantly affected by substance use.^32^ Additionally, Tarekegn et al emphasized the connection between substance abuse and quality of life, suggesting that attention to the mental health of adolescents can serve as a preventive factor in reducing the inclination towards substance use.^26^ The research by Amini and Heidary also indicates that psychological factors such as feelings of security and belonging influence addiction tendencies,^9^ while some studies, like that of Azarmehr and Ahmadi (2019), focused solely on individual and psychological factors such as attention control and anxiety sensitivity, potentially overlooking other important dimensions like social support or economic status.^10^ In fact, research suggests that the combination of aggression, assertiveness, and depression can facilitate the predisposition to addiction, although cultural and social differences between societies must be taken into account.

 In the study by Mansouri Jalilian and Yazdanbakhsh, the tendency toward substance abuse was examined based on self-differentiation and metacognitive beliefs among university students. While their instrument demonstrated acceptable reliability (Cronbach’s alpha), the psychometric evaluation was limited to basic correlational and predictive analyses, without addressing advanced aspects such as construct validity or factor analysis. Their findings indicated that certain metacognitive components and self-differentiation were significant predictors of addiction tendency.^14^ In contrast, the present study employed a comprehensive psychometric approach, thoroughly validating the Addiction Tendency Questionnaire for children through assessments of face, content, construct (exploratory and confirmatory factor analyses), convergent and discriminant validity, as well as reliability and SEM. This instrument not only demonstrated strong reliability and validity but also identified three key factors—ESS, family, and physical-psychological influences—highlighting the prominent role of the family in preventing addiction tendency. Therefore, compared to previous research, the present study offers a more robust and precise tool through advanced statistical and psychometric analyses, providing a stronger foundation for targeted preventive interventions.

 The results of this study indicate that ESS factors play a key role in reducing the potential to use substances, with regression weights for questions Q15 (0.444) and Q19 (0.683) confirming the importance of these variables in the overall analysis. These findings align with Yang et al research, which shows that social support and positive emotions can mitigate the negative impacts of substance use disorders; thus, enhancing positive emotions among adolescents could be an effective strategy in preventive programs.^33^

 In a research entitled “Life skills training Impact on self-mastery and attitude towards drug use in high school students” by Habibi-Kaleybar et al studied a sample size of 30 girls in Tabriz, who applied the questionnaire of Wade and Butcher (1990) for validation. However, Cronbach’s alpha was utilized to measure reliability, nevertheless, they did not mention any validity measurement. The results showed that the addiction tendency questionnaire does not have construct validity. The open test criterion is 0.60, which is likely to separate non-addicted and addicted teenagers from each other. It similarly has a high Cronbach’s alpha.^15^ Cronbach’s alpha report may not be satisfied with being optimal or bad and its value must have highlighted, accordingly. However, in the present study, the reliability index of the measurement and its value was reported as 0.87. Furthermore, the open-ended test criterion reported (0.6) does not have an optimal influence to confirm the reliability, although our results showed that ICC holds a value of 0.88. Correspondingly, quantitative, and qualitative measurement of formal and content validity was performed. On construct validity, four factors were extracted through EFA, all items had high factor loadings, and the extracted factors justified 40% of the items’ variance. In the present study, unlike the other study, the instrument was validated in both genders.^15^ In comparison to the study by Baharvand et al, where only 3.45% of students scored above average, our results show stronger relationships between the variables. This discrepancy may be due to cultural differences, socioeconomic status, or sample size. Their study utilized a descriptive survey method, which may have less accuracy compared to analytical methods like SEM.^34^ Various studies employing CFA and SEM have yielded different results regarding the impacts of various factors on addiction tendencies. For instance, Fathiandastgerdi et al demonstrated that self-efficacy and coping skills predict substance use behavior.^35^ Kaboli and Kiani also highlighted the mediating role of social support and self-efficacy in the relationship between religious inclination and addiction potential, which may differ from our findings that emphasize the importance of family.^36^ Research by Dash et al indicates that personality traits such as neuroticism are associated with substance use.^37^ Additionally, the study by Bunu et al identified peer pressure and lack of parental care as significant factors influencing substance abuse.^38^ However, John et al examined the connection between bicultural identity integration and permissive beliefs about substance use, which contrasts with our findings regarding the positive impact of family, suggesting that adolescents under social pressures are more prone to substance use.^39^ In comparison to the study by Mutamed et al, our results indicate a greater influence of individual, social, and familial factors on addiction tendencies.^40^ The study by Alhyas et al focused solely on adolescents’ perceptions of substance use, potentially overlooking other important dimensions.^31^

## Strengths, Limitations, and Implications

 This study represents the first comprehensive effort in Iran to develop and validate an instrument for assessing addiction tendencies, utilizing advanced psychometric methods such as CFA and SEM. Unlike most studies that utilize a single method to determine the reliability of tools, this study employed multiple methods and various indices toward this goal, which can assist researchers and policymakers in more effectively measuring addiction tendencies in target groups and obtaining more credible results.

 Despite its strengths, several limitations should be acknowledged. First, the sensitive nature of addiction and the associated social stigma posed challenges in participant recruitment and may have introduced selection bias, as individuals willing to participate might differ systematically from those who declined. Second, although multiple psychometric techniques were employed to enhance the precision and reliability of the findings, the cross-sectional design and the sampling strategy may limit the generalizability (external validity) of the results beyond the study population. Third, the multiplicity of analyses, while providing a robust evaluation, increases the risk of type I error and may introduce imprecision in interpreting some associations.

 To address these limitations, future research should aim to replicate these findings in larger and more diverse samples, including different age groups and regions, and consider longitudinal designs to examine causality and stability over time. Additionally, further validation of the tool in various cultural and social contexts is recommended to strengthen its applicability. Also, it is recommended that this tool is administered to different age groups.

 From a practical perspective, the validated instrument developed in this study provides researchers, policymakers, educators, and the Drug Control Headquarters with a reliable means to assess addiction tendencies. Its use can inform the design and implementation of targeted prevention programs and evidence-based policy decisions, ultimately contributing to more effective interventions in the field of addiction prevention.

## Conclusion

 This research focused on the development and validation of a questionnaire assessing addiction tendencies among Iranian children aged 12 to 18 years, demonstrating that this tool possesses desirable validity and reliability. It is suitable for use by relevant organizations such as the Ministry of Education, Welfare Organization, Drug Control Headquarters, and law enforcement agencies, as well as researchers in the field of addiction tendencies among children. The results of the analyses emphasize that the designed questionnaire can serve as a credible tool for measuring addiction tendencies in children. The findings also highlight that family functioning and positive emotions play a key role in reducing addiction tendencies. Given the importance of psychometrics in social research, this study can provide a solid foundation for developing preventive programs within the community. Furthermore, the findings can assist policymakers in designing effective evidence-based programs to reduce substance use among children. Ultimately, the emphasis on the validity and reliability of this tool enhances its significance in future research and clinical interventions.

## Competing interests

 The authors have no conflict of interest to report.

## Data Availability Statement

 The datasets utilized and/or analyzed in the present study can be obtained from the corresponding author upon reasonable request.

## Ethical Approval

 This research received approval from the Ethics Committee of Shiraz University of Medical Sciences with the code IR.SUMS.SCHEANUT.REC.1402.112. Additionally, it obtained endorsements from the Drug Control Headquarters of Iran, the Scientific-Research Committee of the Education Department of Fars Province, and the authorities and security personnel of the Education Department in Fars province and the four educational districts of Shiraz. Oral consent was obtained from each participant. Participants were also assured that their participation in the study was voluntary and that the findings would be reported and published anonymously. To maintain confidentiality, no names or addresses were recorded on the questionnaires. Each questionnaire was assigned a unique code to avoid errors in data collection and related analyses.
